# Supplementary material for: Discovery of genomic intervals that underlie nematode responses to benzimidazoles
Source: PLoS Negl Trop Dis. 2018 Mar 30;12(3):e0006368. doi: 10.1371/journal.pntd.0006368 (PMC5895046; doi:10.1371/journal.pntd.0006368)
Supplement: S1 Fig — Assay detailed in the Materials and Methods section. (PDF) [file pntd.0006368.s001.pdf]

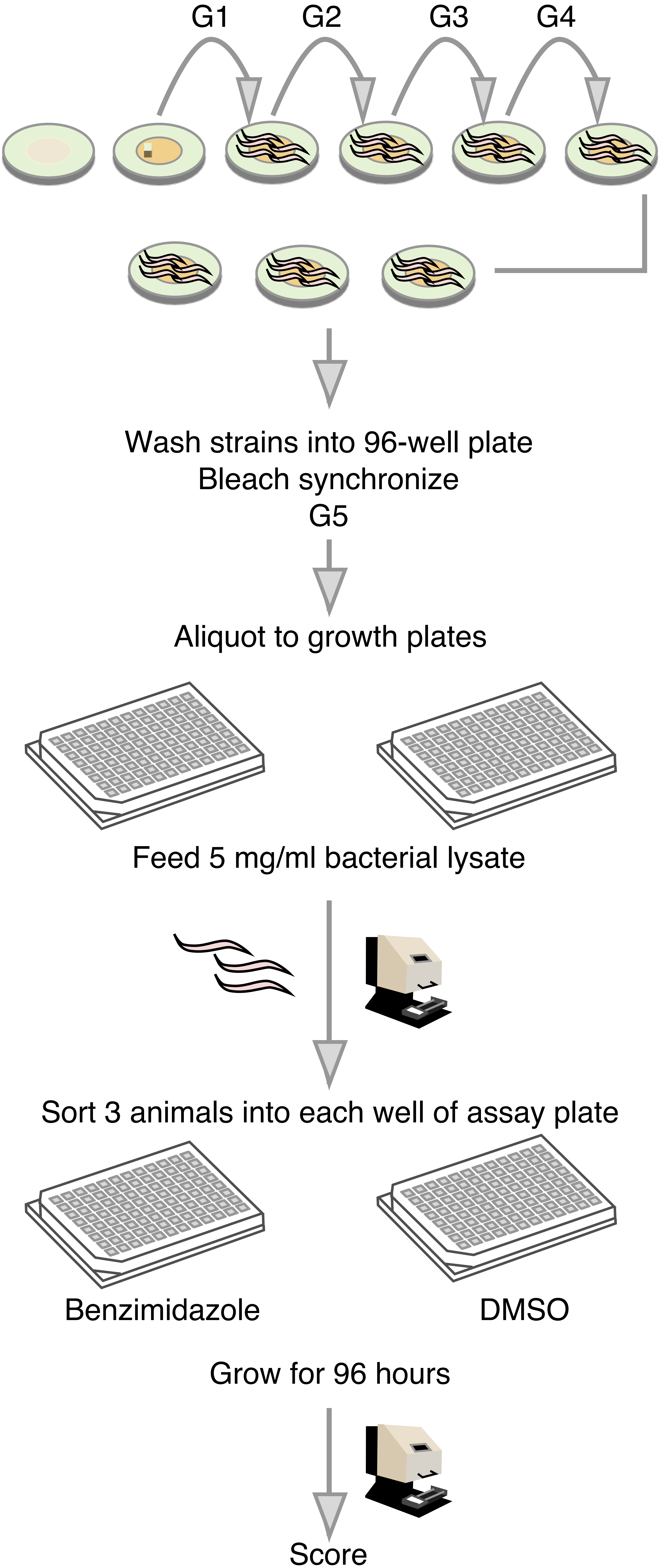
G1
G2
G3
G4
Wash strains into 96-well plate
Bleach synchronize
G5
Aliquot to growth plates
Feed 5 mg/ml bacterial lysate
Sort 3 animals into each well of assay plate
Benzimidazole
DMSO
Grow for 96 hours
Score
