# Supplementary figures and images for: Discovery of genomic intervals that underlie nematode responses to benzimidazoles
Source: PLoS Negl Trop Dis. 2018 Mar 30;12(3):e0006368. doi: 10.1371/journal.pntd.0006368 (PMC5895046; doi:10.1371/journal.pntd.0006368)

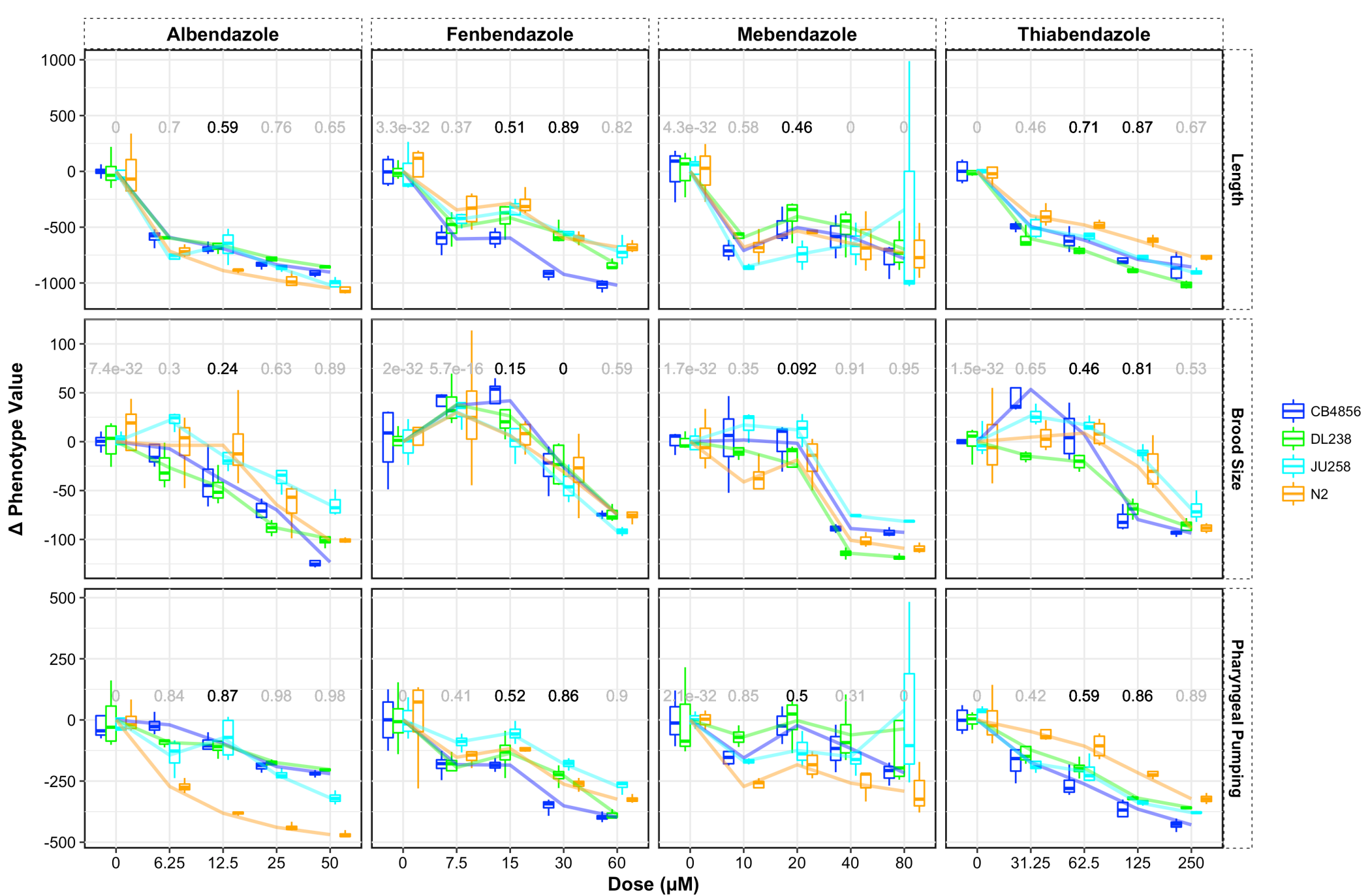

Supplement: S2 Fig — Dose responses were carried out with four genetically diverged strains of C. elegans. Phenotypic responses to four drugs are shown with a representative trait for each primary trait group (length, brood size, and pharyngeal pumping). Heritability values are shown for doses used in subsequent linkage mapping experiments. (PDF) [file pntd.0006368.s002.pdf]

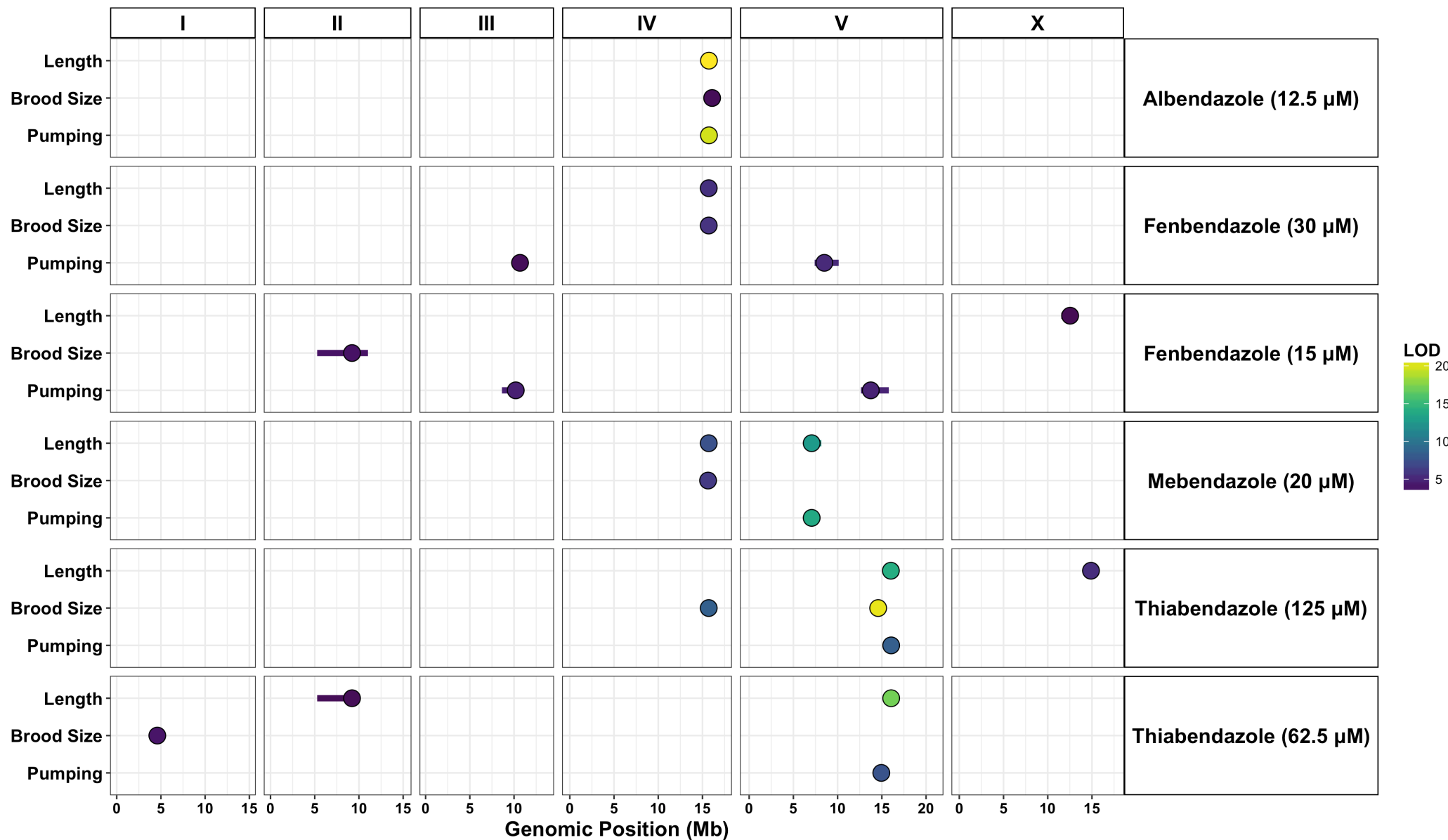

Supplement: S3 Fig — Results of C. elegans linkage mapping experiments are shown for the six drug-dose conditions tested and separated by correlated trait group. QTL peak markers (circles) and confidence intervals (lines) are depicted. Fill color corresponds to the QTL LOD score. Overlapping QTL for a given condition-trait group pair are represented by the trait with the highest significance score. (PDF) [file pntd.0006368.s003.pdf]

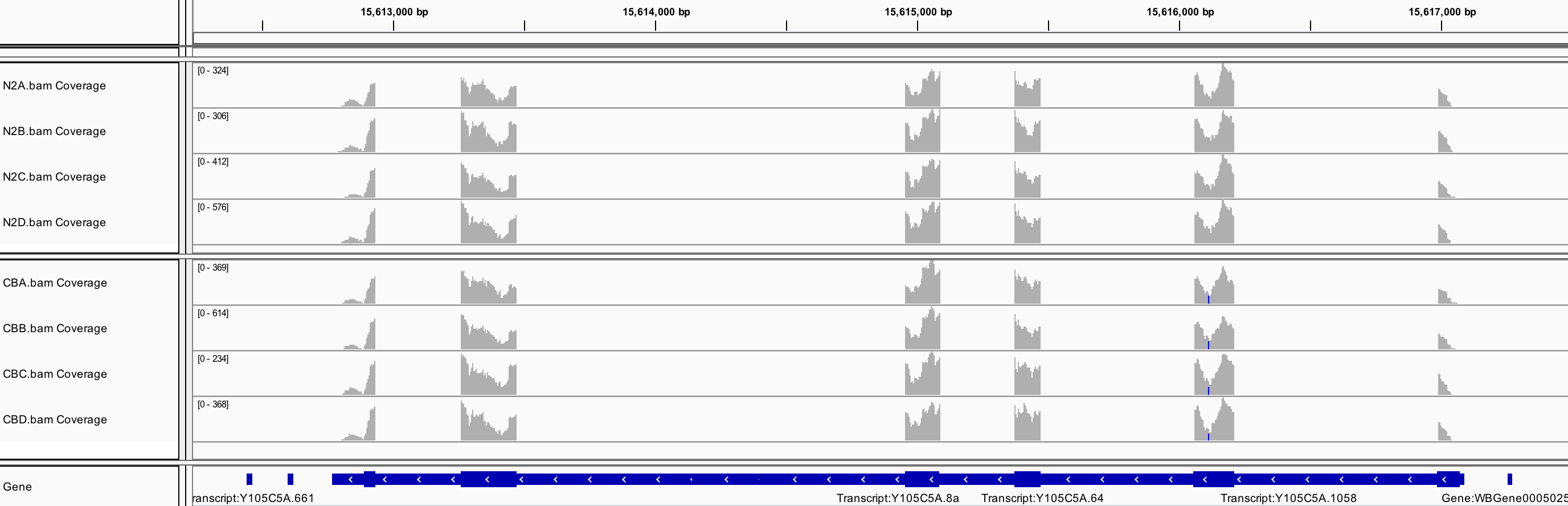

Supplement: S5 Fig — RNA-seq alignment coverage shown for four biological replicates of N2 and CB4856. (PNG) [file pntd.0006368.s005.png]

**A**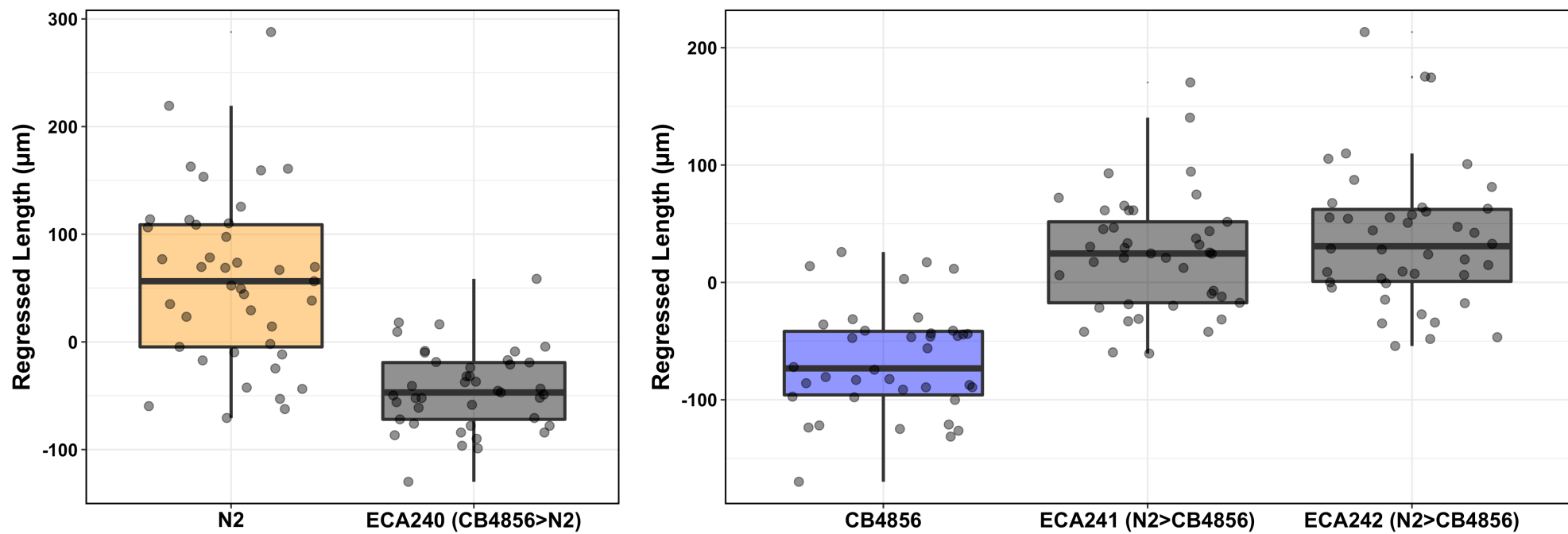**B**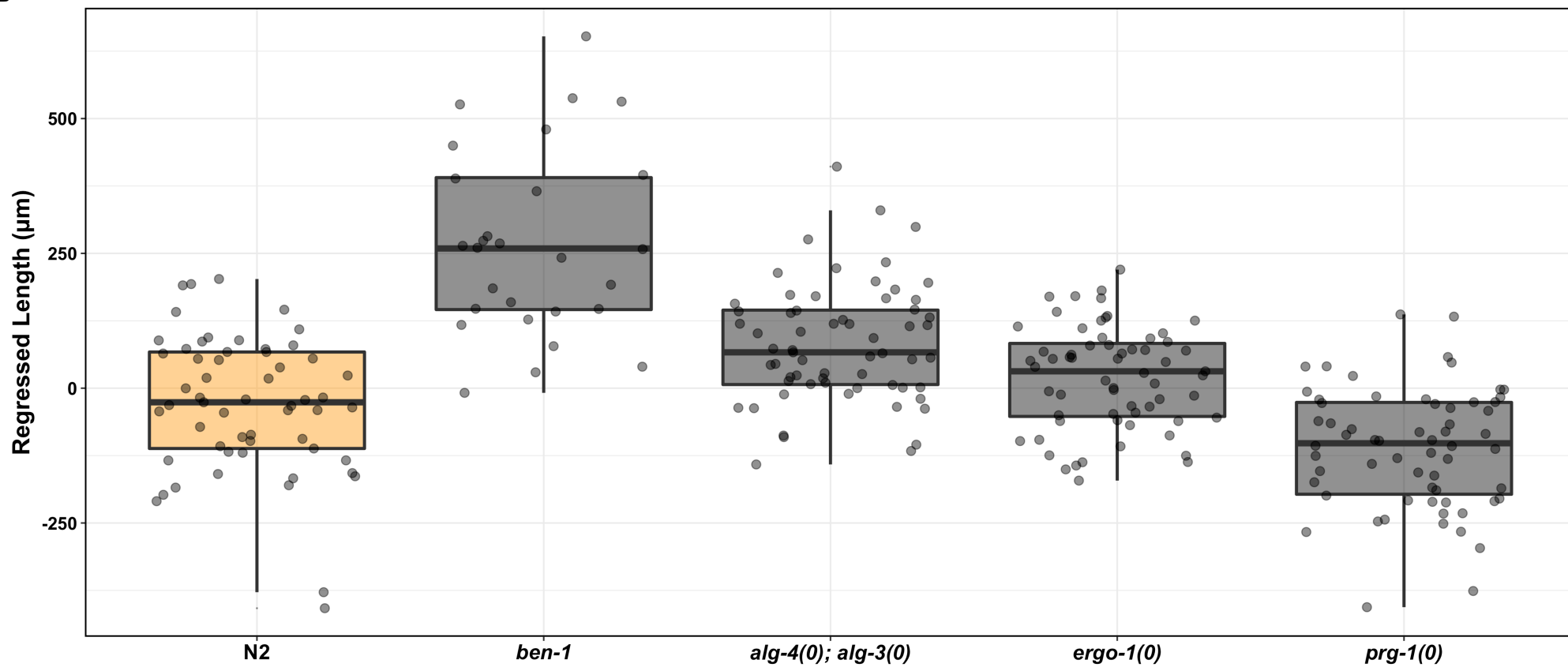

Supplement: S6 Fig — (A) piRNA interval NILs recapitulate QTL direction of effect. Reciprocal NILs covering the primary C. elegans piRNA cluster (chromosome IV: 13.5–17.2 Mb) were phenotyped in the presence of 12.5 μM albendazole. Tukey box plots show drug effects on animal length (75th quantile shown). The introgression of the piRNA cluster from CB4856 into N2 (ECA240) leads to greater albendazole sensitivity compared to N2 (P < 0.001). Introgression of the piRNA cluster from N2 into CB4856 (ECA241 and ECA242) confers greater albendazole resistance compared to CB4856 (P < 0.001). (B) Argonaute mutants that converge on the WAGO 22G RNA pathway were phenotyped in 12.5 μM albendazole. ergo-1 and alg-3; alg-4 mutants, which interact with distinct classes of 26G RNAs, do not confer increased albendazole sensitivity phenotype to the N2 genetic background. Loss of prg-1, the primary Argonaute associated with 21U-RNA/piRNA activity, confers albendazole sensitivity in the N2 background (prg-1(0) vs N2: p < 0.001). However, despite back crossing, the prg-1(0) (prg-1(n4357)) strain exhibited slower growth rate and reduced brood size throughout assay propagation. A known benzimdazole-resistance allele, ben-1, is included as a positive control and basis for comparison of relative effect sizes (prg-1(0) vs ben-1: p < 0.001). Additionally, the magnitude of the difference in albendazole response between N2 and ECA240 (A) is approximately 34% of the difference between N2 and ben-1 (B). Mutations in the parasite homologs of ben-1 are known to result in clinically significant benzimidazole resistance, and this comparison underscores the potential importance of the amount of resistance explained by the major albendazole QTL on chromosome IV. t-tests were used for statistical comparisons (p-values reported in S9 Table). (PDF) [file pntd.0006368.s006.pdf]

A

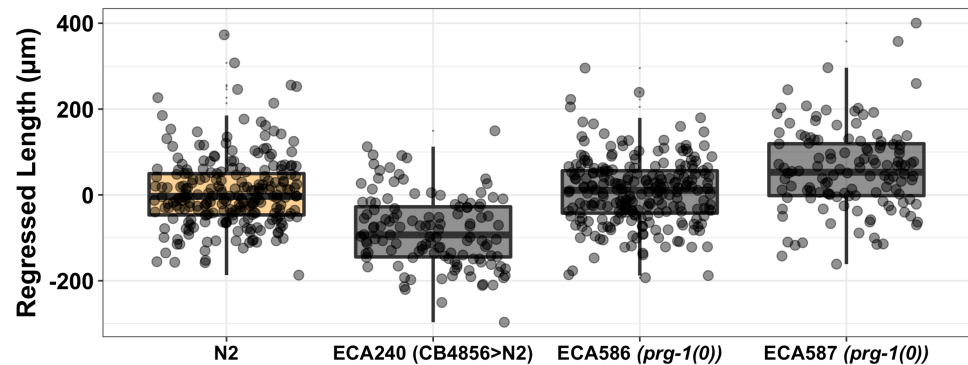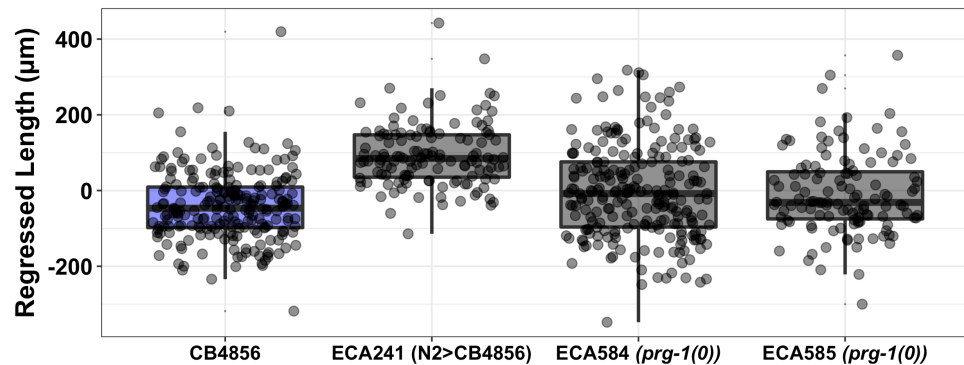

B

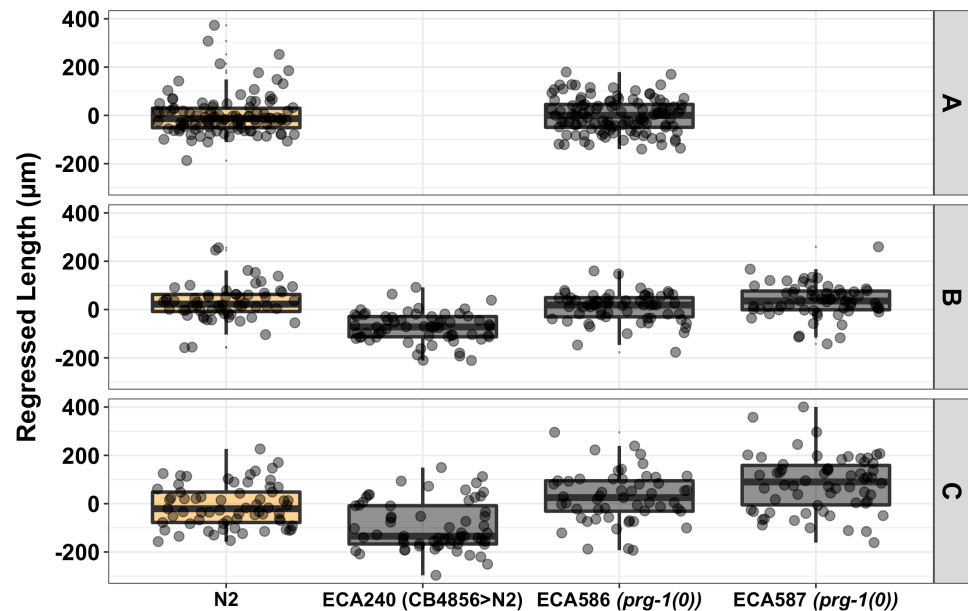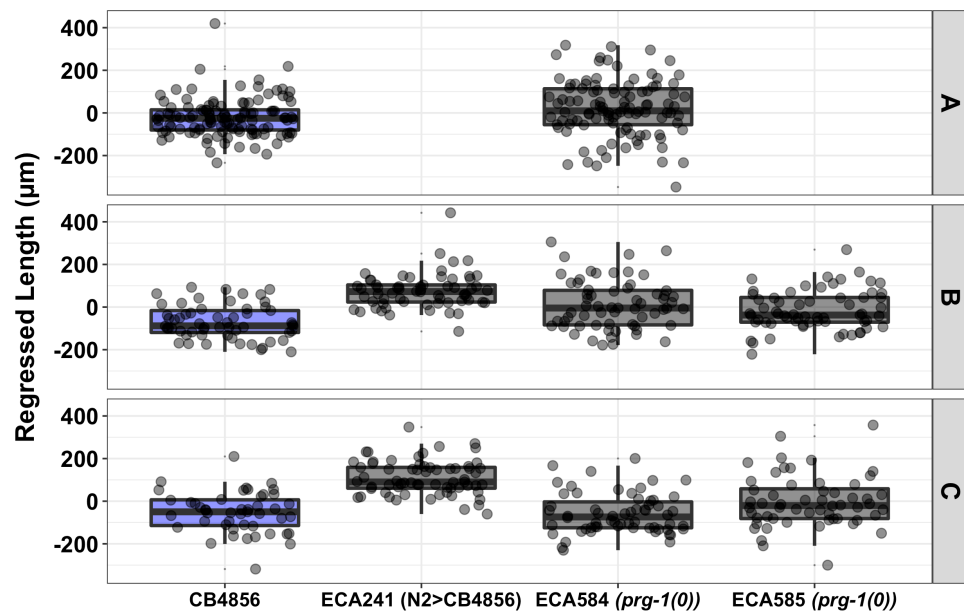

Supplement: S7 Fig — (A) Combined data from three independent replicate assays recapitulate the established NIL effect but do not support the hypothesis that this effect is prg-1 dependent. Tukey box plots show drug effects on animal length (75th quantile shown). Individual assays shown in (B) and one-sided t-tests (alpha = 0.05) were used to test whether prg-1(0) in the N2 background (ECA586 and ECA587) would lead to greater sensitivity compared to N2 (not significant: p = 0.778 for ECA586; p = 0.9997 for ECA587) and that prg-1(0) in the CB4856 background (ECA584 and ECA585) would lead to greater resistance compared to CB4856 (not consistently significant across replicate days). One-tailed t-tests were used for all comparisons (p-values reported in S9 Table). (PDF) [file pntd.0006368.s007.pdf]

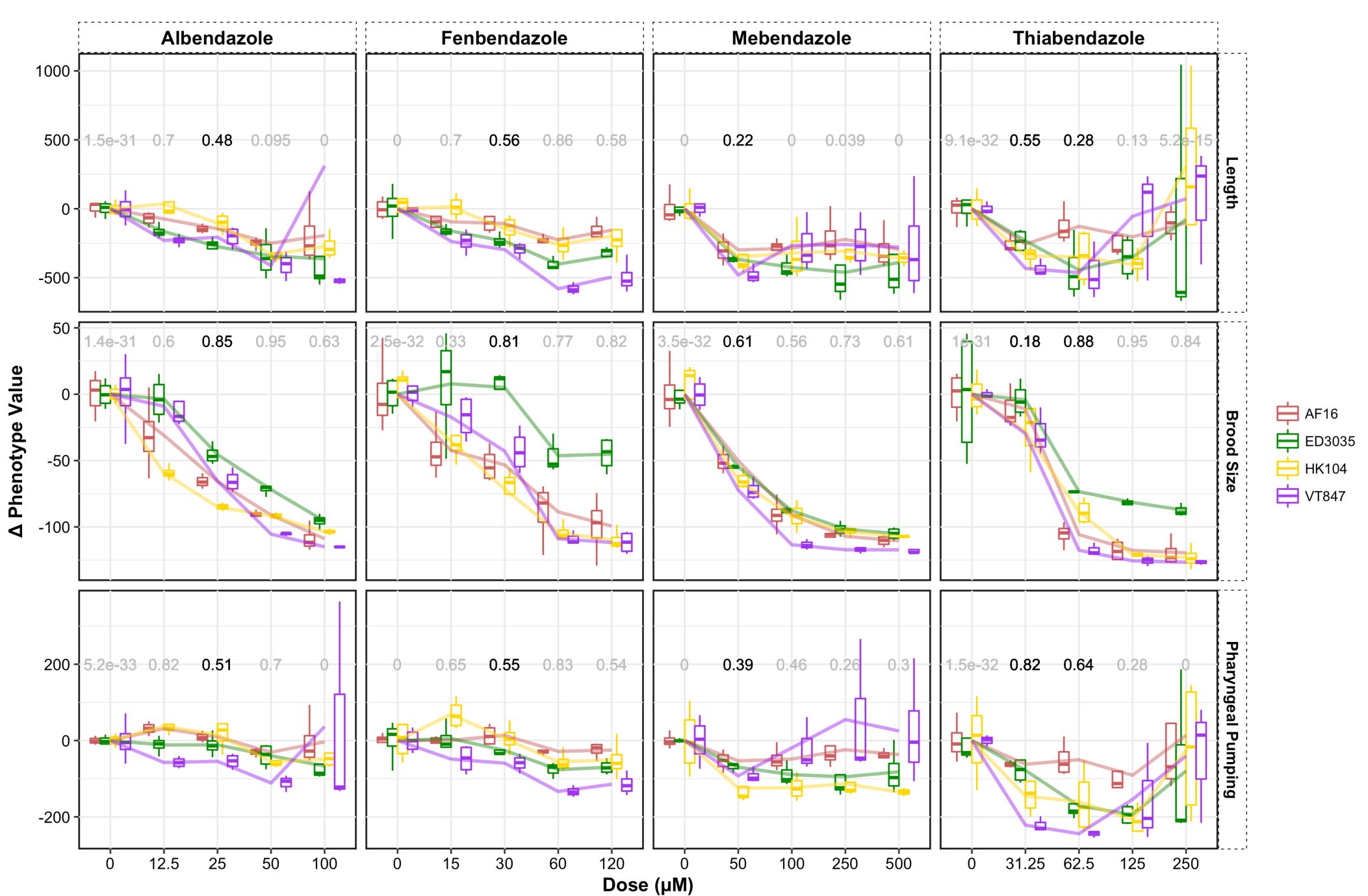

Supplement: S8 Fig — Dose responses were carried out with four genetically diverged strains of C. briggsae. Phenotypic responses to four drugs are shown with a representative trait for each primary trait group (length, brood size, and pharyngeal pumping). Heritability values are shown for doses used in subsequent linkage mapping experiments. For thiabendazole, we selected a linkage mapping dose (40 μM) that falls between the concentrations where heritability is shown. (PDF) [file pntd.0006368.s008.pdf]

Albendazole (25 uM)

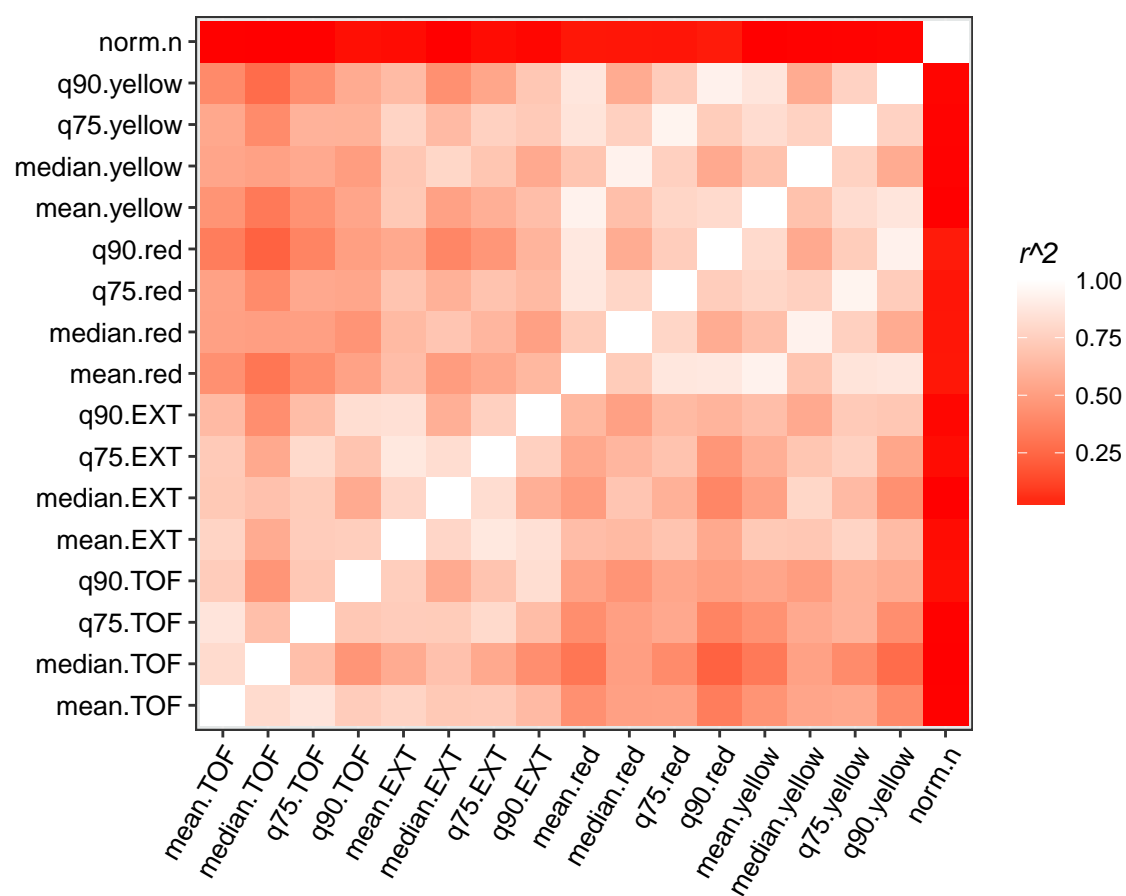

Fenbendazole (30 uM)

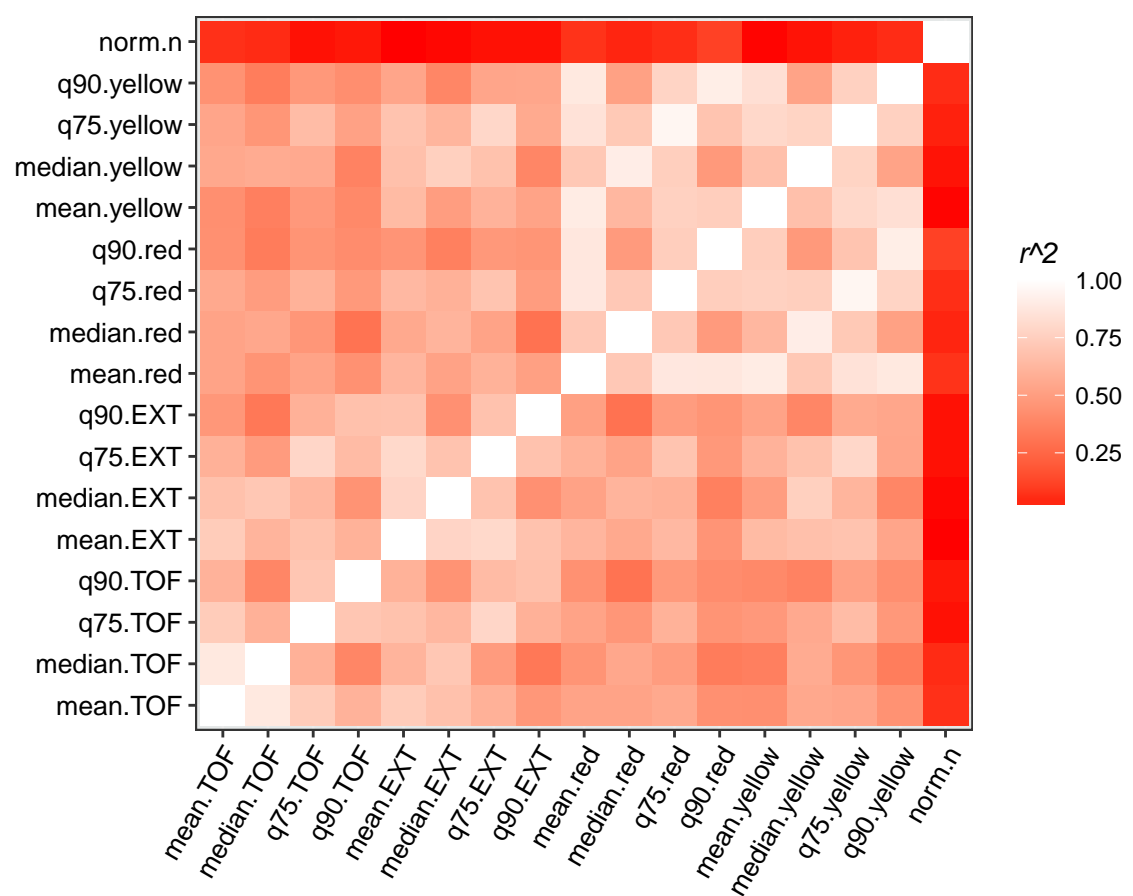

Mebendazole (50 uM)

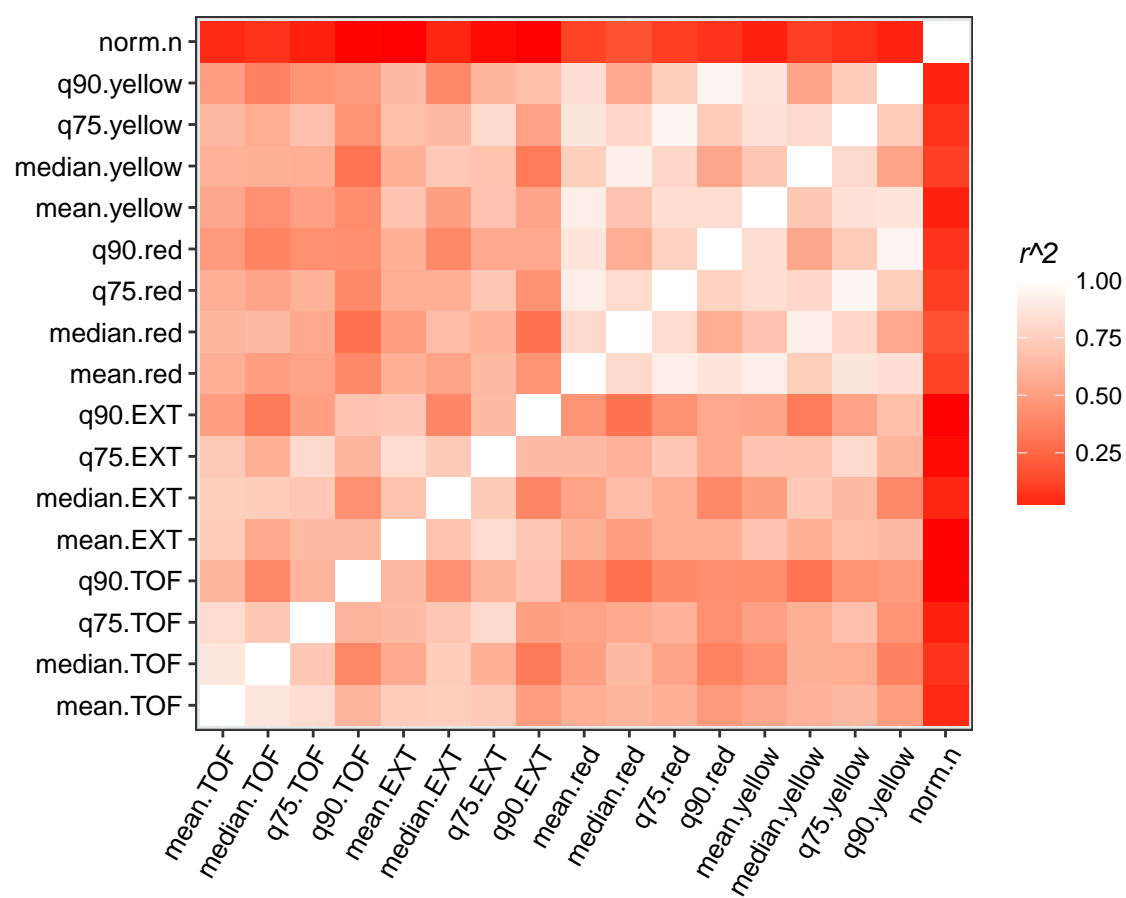

Thiabendazole (40 uM)

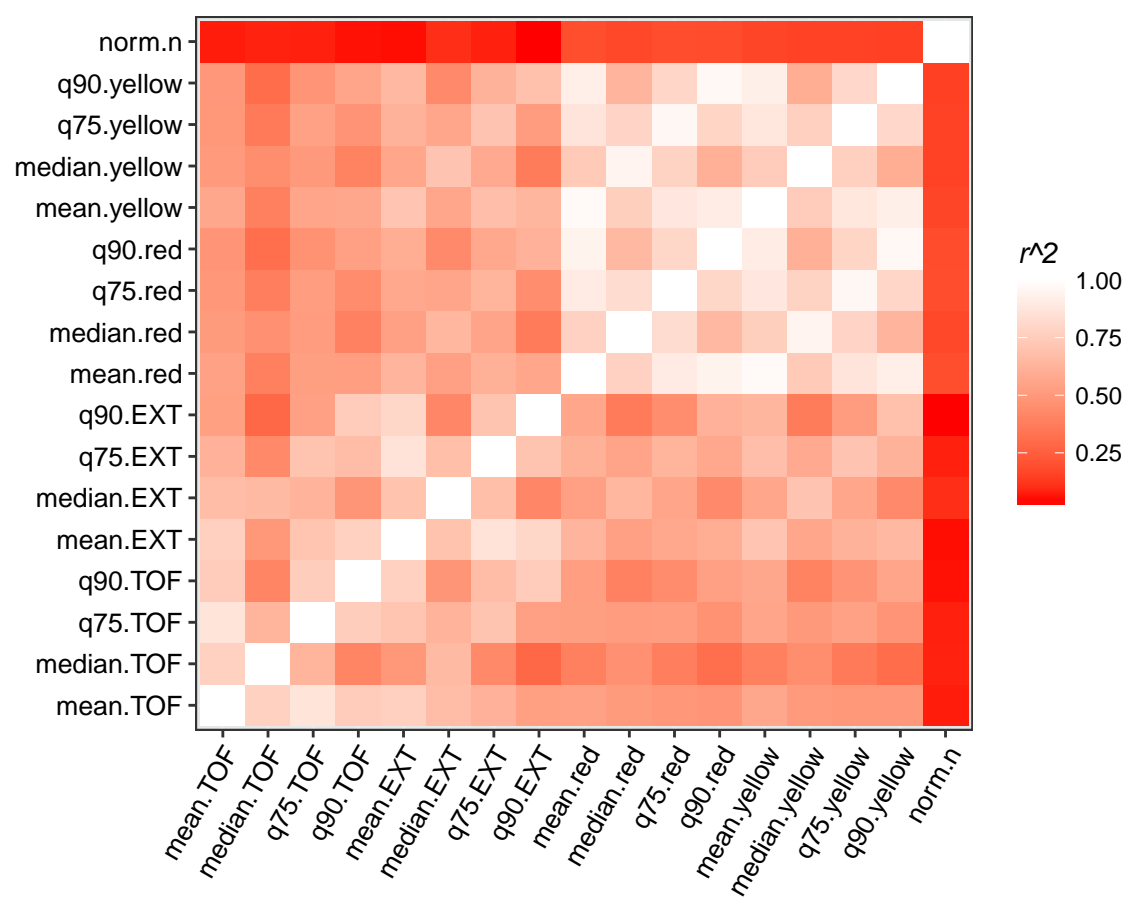

Supplement: S9 Fig — The correlation structure (Pearson’s correlation coefficient) of summary statistics for measured parameters of animal size (time-of-flight (TOF) and optical density (EXT)), pharyngeal pumping (red and yellow fluorescence), and brood size (norm.n) are shown for each drug tested. (PDF) [file pntd.0006368.s009.pdf]

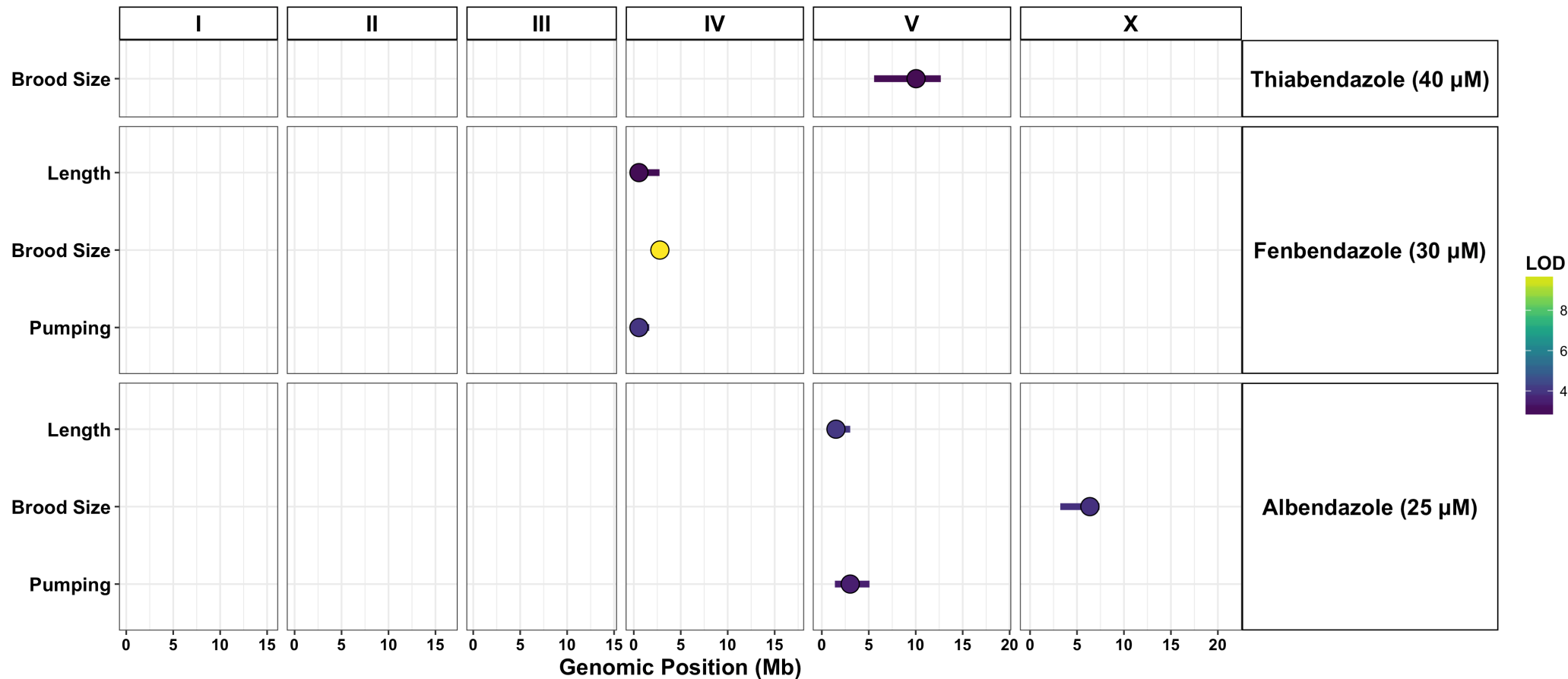

Supplement: S10 Fig — Results of C. briggsae linkage mapping experiments are shown for the drug-dose conditions tested and separated by correlated trait group. QTL peak markers (circles) and confidence intervals (lines) are depicted. Fill color corresponds to the QTL LOD score. Overlapping QTL for a given condition-trait group pair are represented by the trait with the highest significance score. (PDF) [file pntd.0006368.s010.pdf]
