# Supplementary material for: Discovery of genomic intervals that underlie nematode responses to benzimidazoles
Source: PLoS Negl Trop Dis. 2018 Mar 30;12(3):e0006368. doi: 10.1371/journal.pntd.0006368 (PMC5895046; doi:10.1371/journal.pntd.0006368)
Supplement: S4 Fig — The correlation structure (Pearson’s correlation coefficient) of summary statistics for measured parameters of animal size (time-of-flight (TOF) and optical density (EXT)), pharyngeal pumping (red and yellow fluorescence), and brood size (norm.n) are shown for each drug and dose combination used in linkage mapping. (PDF) [file pntd.0006368.s004.pdf]

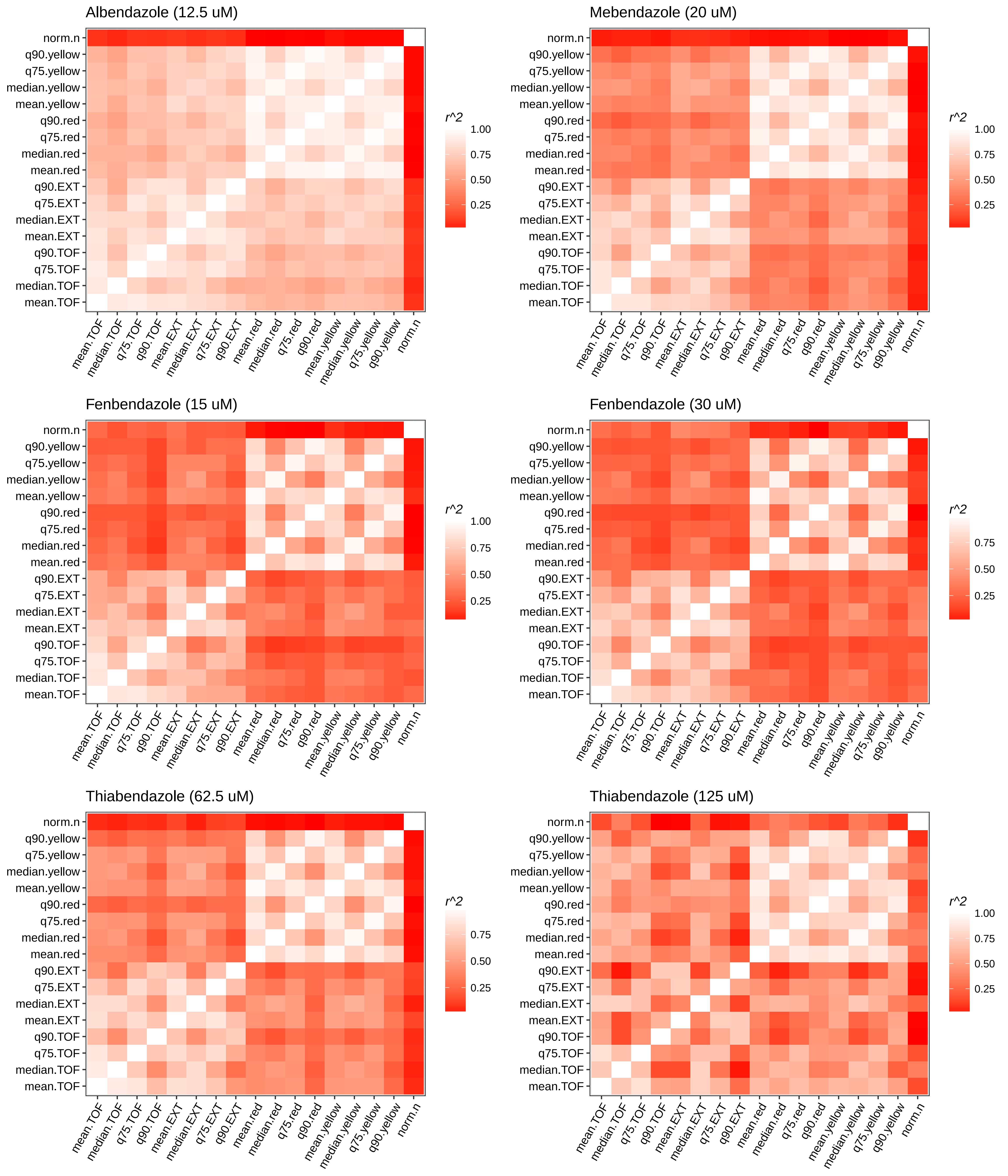
Albendazole (12.5 uM)
norm.n
q90.yellow
q75.yellow
median.yellow
mean.yellow
q90.red
q75.red
median.red
mean.red
q90.EXT
q75.EXT
median.EXT
mean.EXT
q90.TOF
q75.TOF
median.TOF
mean.TOF
r^2
1.00
0.75
0.50
0.25
Mebendazole (20 uM)
r^2
1.00
0.75
0.50
0.25
Fenbendazole (15 uM)
r^2
1.00
0.75
0.50
0.25
Fenbendazole (30 uM)
r^2
0.75
0.50
0.25
Thiabendazole (62.5 uM)
r^2
0.75
0.50
0.25
Thiabendazole (125 uM)
r^2
1.00
0.75
0.50
0.25
