# Supplementary material for: Discovery of genomic intervals that underlie nematode responses to benzimidazoles
Source: PLoS Negl Trop Dis. 2018 Mar 30;12(3):e0006368. doi: 10.1371/journal.pntd.0006368 (PMC5895046; doi:10.1371/journal.pntd.0006368)
Supplement: S1 Methods — Primers and starting RIAILs used in the construction of whole piRNA-interval NILs. Mutant strains and oligos used to confirm and propagate existing mutant alleles through back-crossing and to construct genome-edited strains. (PDF) [file pntd.0006368.s011.pdf]

C. elegans whole piRNA interval NILs

- piRNA interval: Chr IV: 13.5 - 17.2 Mbs / N2 Size: 526, CB4856 Size: 942
- Left Primers (InDel Left: 13207120)
  - oECA904: aacagatactcgccgttgct
  - oECA905: atttgtaccacgcgtgacct
- Right Primers (InDel Right: 17356993) / N2 Size: 443, CB4856 Size: 596
  - oECA910: gacaacgcccactacgacaa
  - oECA911: acccaaccagttgagcacat
- CB4856>N2 NIL (ECA240) / eanIR160 (IV, CB4856>N2) / Starting RIAIL: QX349
- N2>CB4856 NIL (ECA241) / eanIR161 (IV, N2>CB4856) / Starting RIAIL: QX375

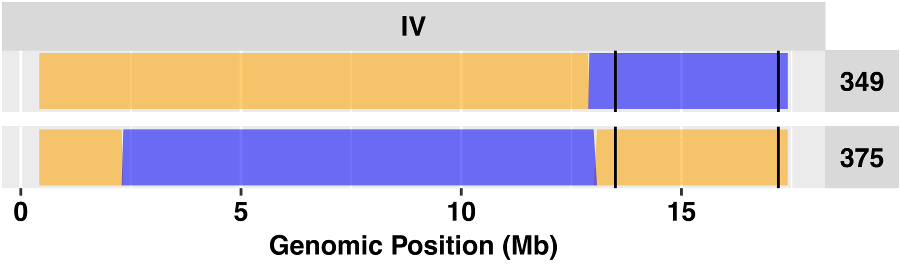

C. elegans mutants

- piRNA mutants confirmed and/or backcrossed in N2 background
  - ECA270: *alg-4(tm1184);alg-3(tm1155)* [ALG-3/4 class 26G RNA]
  - ECA271: WM158 / *ergo-1(tm1860)* [ERGO-1 class 26G RNA]
  - ECA286: *prg-1(n4357)* [21-U RNA]: SX922/ECA269 backcrossed to N2 (9x) and selfed (6x)
  - FX234: *ben-1*
- Mutant confirmation PCR primers
  - *ergo-1(tm1860)*: oECA1033 AAGCGTACGAACCCGAGCTT / oECA1034 GAGCGGCTGCTCAGAAGACT
- *prg-1(n4357)* PCR primers for backcrossing
  - oECA1019: AGTCGTGGTACAGATCGTAG
  - oECA1020: GAGAGGCCGTGGTTCAGGAT
  - Wild-type size = 1972, *prg-1(n4357)* size = 1248 (724 bp deletion)

C. elegans CRISPR *prg-1* loss-of-function strains

- Primers and oligos
  - oECA2002: GUUAGCCUUCGAAUCAACGG
  - oECA2003: CGCUGUGACCGACAAAGCUG
  - oECA2004: GGGTACTATCCAACCCGATCTTTTCATTCG
  - oECA2042: CGCGTTTCGTGACAATGATAAATGCS (3’ wobble to account for variant between N2 and CB4856)
  - *dpy-10* crRNA: GCUACCAUAGGCACCACGAG
  - *dpy-10* repair oligo: CACTTGAACTTCAATACGGCAAGATGAGAATGACTGGAAACCGTACCGCATGCGGT-GCCTA GGTAGCGGAGCTTCACATGGCTTCAGACCAACAGCCTAT
- Strains
  - ECA584: Exon 1-7 deletion (CB4856) and ECA585: Exon 2-7 deletion (CB4856)
  - ECA586: Exon 1-7 deletion (N2) and ECA587: Exon 2-7 deletion (N2)
